# Technological architecture for a multi-region solution within the regulation of Brazil’s Unified Health System

**DOI:** 10.3389/fdgth.2026.1763929

**Published:** 2026-02-20

**Authors:** Pablo Holanda Cardoso, Tiago de Oliveira Barreto, Janaína Luana Rodrigues da Silva Valentim, Karilany Dantas Coutinho, João Paulo Queiroz dos Santos, Antônio Higor Freire de Morais, Nícolas Vinícius Rodrigues Veras, Aldo Eduardo de Almeida Portela, Juliano Silva Melo, Célio da Costa Barros, Andréa Santos Pinheiro, Monise Barros Dantas, Jordana Crislayne de Lima Paiva, José Arilton Pereira Filho, Raul Silva de Almeida, Walkyso dos Santos Júnior, Arthur Meireles da Silva, Elionai Augusto Silva de Melo, Douglas Lemos Inácio da Silva, João Victor Medeiros Crisostomo, Sandra Rubina Freitas Cardoso dos Santos, Claudia Maria Fileno Miranda Veloso, Guilherme Medeiros Machado, Ricardo Alexsandro de Medeiros Valentim

**Affiliations:** 1 3S Saúde, Natal, Rio Grande do Norte, Brazil; 2 Advanced Nucleus of Technological Innovation (NAVI), Federal Institute of Rio Grande do Norte (IFRN), Natal, Rio Grande do Norte, Brazil; 3 Center for Global Studies, Open University of Portugal (CEG-UAb), Lisboa, Portugal; 4 Laboratory of Technological Innovation in Health (LAIS), Federal University of Rio Grande do Norte (UFRN), Natal, Rio Grande do Norte, Brazil; 5 Department of Biomedical Engineering and Postgraduate Program in Health Management and Innovation, Federal University of Rio Grande do Norte (UFRN), Natal, Rio Grande do Norte, Brazil; 6 Secretary of Public Health of Mato Grosso, Cuiabá, Mato Grosso, Brazil; 7 Federal Court of Accounts, Fortaleza, Ceará, Brazil; 8 University Institute of Lisboa (ISCTE), Lisboa, Portugal; 9 Center for Global Studies (ESTGA), Research Unit in Governance, Competitiveness and Public Polices (GOVCOPP), University of Aveiro, Aveiro, Portugal; 10 LyRIDS, ECE-Engineering School, Paris, France

**Keywords:** Brazilian Unified Health System, health systems, regulation, technological architecture, transparency in healthcare

## Abstract

**Introduction:**

This article presents the design and implementation of a digital health technology architecture focused on healthcare regulation in Brazil's National Health System (SUS). The objective was to develop an architectural model capable of optimizing resource allocation, increasing transparency, and integrating health information from different levels of care, with a focus on reducing inequalities in access.

**Methods:**

Methodologically, a transdisciplinary applied research approach based on action research was adopted, with iterative development cycles in accordance with agile methodologies. The architecture was implemented in the states of Rio Grande do Norte, Espírito Santo, and Mato Grosso, respecting regional specificities and integrating international interoperability standards, as well as architectural principles and software engineering.

**Results:**

The results point to flexibility, interoperability, real-time monitoring, queue management, and transparency, including direct access for control bodies and process auditability.

**Discussion and Conclusions:**

It can be concluded that the proposed architecture represents a significant advance for equity in access and could serve as a basis for solutions on a national and international scale.

## 1 Introduction

In Brazil, public healthcare is provided by the Brazilian National Health System (SUS), which is considered one of the largest and most complex healthcare systems in the world, facing challenges inherent to its scale and the socioeconomic diversity of the population it serves. In general, the SUS faces several challenges related to healthcare management, resource allocation, quality of care, and access regulation, especially about ensuring equitable and timely access to healthcare services in a context of limited resources and growing demands [1–3].

Healthcare regulation was established by Ordinance No. 1,559, which enacted the National Policy for Regulation of the SUS, and was consolidated by Ordinance No. 28/2017, to organize, coordinate, and optimize the population’s access to health services offered under the SUS. This policy seeks to ensure that all citizens, regardless of their geographical location or socioeconomic status, have equitable and efficient access to the necessary care, respecting the fundamental principles of the SUS, such as universality, equity, and comprehensiveness [4–6].

However, it should be noted that the healthcare regulation process still faces shortcomings that directly affect the quality and speed of care. They are mainly linked to the disorganization of flows, the lack of integration between systems, and the technological limitations of regulation centers. One of the main problems is the difficulty in quickly and accurately viewing the availability of resources, such as hospital beds, access to specialists, or tests. Many decisions are made without using any information system that could guide the correct deliberation [7, 8].

Furthermore, there is a lack of automated and standardized criteria within the care process, which makes the regulatory process time-consuming and often dependent on the subjective assessment of each professional. As protocols vary between different regions and levels of healthcare, many cases end up being decided unfairly, such as, for example, the care of certain groups to the detriment of others that would be a priority, which violates the principle of equity of the SUS. Another critical issue is the lack of communication between the systems used by municipalities and states. Instead of working in an integrated manner, each location tends to adopt its own tools, which leads to duplicate records, loss of information, and difficulty in tracking patient history in the public health system [8–10].

Another problem is associated with the insufficient supply of efficient computerized systems and poor assistance services, which promotes increased judicialization in services. This dynamic generates a paradoxical effect because, in seeking to guarantee individual care through court decisions, there is a distortion in the order of queues and regulatory priorities established by the SUS. This scenario compromises equity in access, favoring users who are able to resort to the courts over those who wait in the regular queue, in addition to implying abrupt changes in the planning and allocation of public resources. The excessive number of lawsuits overloads the system, which further impairs the flow of care and undermines the efficiency of health regulation [11, 12].

The disorganization of the health care network in Brazil is similarly manifested by problems in the flow between different levels of care and by the lack of integration between health care units. This fragmentation compromises continuity of care, causing delays and difficulties in patient referrals, as well as overburdening specific sectors, such as specialized care. The decentralization of the SUS, while expanding access, has also led to the existence of isolated local systems with little regional coordination. The lack of integration and effective communication between levels of care results in fragmented, redundant, or even unnecessary care, which undermines the efficiency of the system and the guarantee of effective and continuous care for users. This disorganization compromises the effectiveness of the universality and comprehensiveness envisaged by the SUS, exacerbating inequalities in access and quality of service provided [13, 14].

Another challenge is to drive and maintain transparency among the systems developed for public health. Although essential, many care processes, including regulation, have difficulty making their processes transparent because they are, in many situations, manual and not very computerized. Fraga and Lira (33) point out that the transparency process is essential for maintaining clear, supervised assistance flows and promoting social justice, given that this allows the population to more clearly identify the progress of service and regulation queues, as well as the existing priority criteria. This process minimizes suspicions of fraud, embezzlement, or manipulation and increases the sense of fairness in access.

In Brazil, the regulatory system adopted by the Ministry of Health is SISREG, developed by the SUS IT Department (DataSUS) starting in 1999 to manage outpatient and hospital care. Its most updated version is SISREG III, developed in mid-2006. Although SISREG has consolidated itself as a consistent initiative for health regulation, its architecture is obsolete, built as a highly coupled monolith, lacking integration with other government platforms, transparency, and monitoring capacity. For a considerable portion of healthcare, SISREG contributes to a decline in service quality due to difficulties in information management [15, 16].

Despite the widespread adoption of digital solutions for health regulation, a significant gap persists in the definition of interoperable, auditable, and scalable software architectures capable of supporting decentralized and multi-regional regulatory processes within public health systems. The existing solution, SISREG, is predominantly based on a monolithic format that does not even allow for local customization, hindering interoperability with other health systems, limiting the traceability of regulatory decisions, and restricting transparency and governance at the systemic level. From a scientific point of view, this scenario highlights the lack of reference architectural models that explicitly address these challenges while remaining adaptable to heterogeneous organizational and regulatory contexts.

Given this scenario, the proposal for a digital health technology architecture emerges as a tool to modernize and optimize health regulation within the SUS, aiming to improve the efficiency, transparency, and quality of services provided. This study focuses specifically on the design and validation of a reference architecture for health regulation, capable of adapting to different regional needs, ensuring data integration in accordance with international standards. The system implemented in this work serves as an instantiation of the proposed architecture, supporting its validation in real-world contexts, rather than constituting the study’s main contribution, thus strengthening the Brazilian Unified Health System through an architectural approach.

## 2 Sociodemographic and health regulation characteristics of implementation sites

The regulatory architecture has already been implemented in three different Brazilian states: Rio Grande do Norte, Espírito Santo, and Mato Grosso. In order to present the scope of the implementation sites, this section presents the sociodemographic characteristics of the sites in terms of health, as well as their similarities and differences in terms of health regulation.

### 2.1 Rio Grande do Norte

Rio Grande do Norte (RN), located in the Northeast region of Brazil, has a wide social, economic, and demographic diversity. According to recent data from the IBGE [17], the state has approximately 3.3 million inhabitants spread across 167 municipalities and covers an area of 52,811 km${}^{2}$. The capital Natal is the most populous municipality, home to more than 750,000 people, followed by Mossoró and Parnamirim, which have populations of approximately 265,000 and 253,000, respectively. RN has a high urbanization rate of 77.8% and an average population density of 62.5 inhabitants per square kilometer, concentrated mainly in coastal areas.

In the area of public health, the state is organized into eight health regions, each with a reference municipality that coordinates the regulation of health services in the respective area. São José de Mipibu is the hub of the first region, while Mossoró, João Câmara, Caicó, Santa Cruz, Pau dos Ferros, Natal, and Assu, respectively, lead the others, with Assu representing the eighth region [4, 18].

According to data from the National Registry of Health Establishments (CNES) and the TabNET platform, Rio Grande do Norte has 6,173 health facilities. Of these, 1,326 are basic municipal units, 10 are Emergency Care Units (ECUs), and 21 are state hospitals. Hospitals are mainly concentrated in the regions of Natal (seventh region) and Mossoró (second region), and this centralization may affect access to specialized services for the population in rural areas.

### 2.2 Espírito Santo

The state of Espírito Santo (ES) is located in the southeastern region of Brazil and has diverse demographic and socioeconomic characteristics. According to the Brazilian Institute of Geography and Statistics [17], the state has an estimated population of approximately 3.8 million inhabitants distributed across 78 municipalities, occupying a territorial area of approximately 46,074 km${}^{2}$. The most populous city is Serra, with approximately 537,000 inhabitants, followed by Vila Velha, Cariacica, and Vitória, the latter being the state capital. The population density of Espírito Santo is approximately 83 inhabitants per square kilometer, being higher in metropolitan areas.

With regard to the organization of the public health system, Espírito Santo also adopts a regionalized model, divided into three health regions: Central North (with 29 municipalities), Metropolitan (with 23 municipalities), and South (with 26 municipalities). As in RN, this structure aims to decentralize services and improve access for the population in different regions of the state.

### 2.3 Mato Grosso

Mato Grosso (MT), located in the Central-West region of Brazil, has a large land area and a continuously growing population. With an area of approximately 903,208 km${}^{2}$, it is the third largest state in the country in terms of land area. According to data from the Brazilian Institute of Geography and Statistics [17], the state has approximately 3.6 million inhabitants spread across 142 municipalities. The capital, Cuiabá, is the most populous municipality, with around 650,000 inhabitants, followed by Várzea Grande, Rondonópolis, and Sinop. The population density of Mato Grosso is low, around 4.05 inhabitants per square kilometer, reflecting its vast, sparsely populated area, especially near the Amazon region.

The state’s healthcare regionalization is divided into six macro-regions (center-northwest, center-north, north, east, west, and south) and 16 healthcare regions: Cuiabá lowlands (14 municipalities), South (19 municipalities), garças araguaia (10 municipalities), West (12 municipalities), Northwest (7 municipalities), Lower Araguaia (7 municipalities), Teles Pires (14 municipalities), Mid-North (10 municipalities), North-Central (7 municipalities), Upper Tapajós (6 municipalities), Vale do Arinos (4 municipalities), Vale do Peixoto (5 municipalities), Mid-Araguaia (8 municipalities), Southwest (10 municipalities), North (6 municipalities), and North Araguaia Karajá (5 municipalities). Unlike the other states mentioned, Mato Grosso seeks to increase the number of regional distributions, maintaining a smaller number of municipalities and promoting greater local control.

### 2.4 Similarities and differences in the regulatory process

Understanding the similarities and differences between regulatory processes is essential to endorsing the development of an architecture that can encompass the specific characteristics of each region. In this sense, there are three different Brazilian states that, although they have decentralized state regulation in health regions, have different dissociation profiles. While Espírito Santo concentrates a larger number of municipalities in only three health regions, Mato Grosso does the opposite, expanding the number of health regions with a smaller number of municipalities, and Rio Grande do Norte has a more balanced division between the number of municipalities and regions.

As for similarities, all three states have complete state management of the services for which they are responsible. That is, for the main health services offered, the request and provision of the service are coordinated by a state regulation center and are carried out by state health units, contracted with private units or units in other municipalities. All three states previously used the national regulation system SISREG; however, due to its limitations, obsolescence, lack of integration, and lack of transparency, it often does not meet all the needs of a dispatch center [15, 18, 19].

As for differences, in the context of outpatient regulation, Espírito Santo has adopted the Territorial Formative Self-Regulation (ARFT) model since 2020, a regulatory mechanism that aims to bring primary health care closer to specialized care, so that medical specialists can care for patients in conjunction with primary care [20]. Primary health care units submit requests to specific hospitals to ensure the maintainability of ARFT. Another peculiarity of ES is the inclusion of teleconsultations as a mechanism for bringing PHC closer to specialties and reducing waiting lists. In RN, the regulation process occurs through contracts in which a request from a basic health unit can be directed to any specialized health provider.

In the case of Mato Grosso, regulation is characterized by agreements between municipalities with a decentralized structure in which only some procedures are managed directly by the state. As mentioned earlier, the state is divided into 16 health regions, with regulatory centers located in each municipality, and it is these centers that manage the process locally. Furthermore, the MT adopts telemedicine as a support tool, allowing patients to go to primary care units and, if necessary, be treated by specialized hospitals. It is worth noting that any municipal hospital is permitted to meet this demand for telemedicine, expanding access and coverage of the regulation in the state.

## 3 Materials and methods

### 3.1 Methodological approach to the research

The design of the digital technology architecture for health regulation was conducted as applied research, of a transdisciplinary nature, based on the action-research methodology. This approach was chosen because it allows for the simultaneous development and evaluation of technological solutions in real-world contexts, involving health managers, healthcare professionals, information technology teams, and researchers in the field of digital health, with continuous stakeholder participation throughout the entire investigation and development process, specifically to support the co-design, improvement, and contextual validation of the proposed architectural model.

The adopted method integrated principles of software engineering, implementation science, and digital governance in health, seeking to align technical rigor and social relevance. Thus, each stage of the process, from requirements gathering to artifact validation, was carried out collaboratively and iteratively to ensure that architectural decisions reflected the operational needs of healthcare units (requesters and providers), regulatory centers, health secretariats, and all other entities involved, as well as national health regulation guidelines. The requirements gathering phase involved healthcare managers, physicians, nurses, and technicians, and was explicitly oriented towards identifying the architectural limitations of the previously used system, particularly those related to information fragmentation, interoperability, traceability, and the efficiency of regulatory processes.

The methodological framework was based on iterative cycles of design, development, and validation, aligned with the SCRUM agile development methodology, formally applied, with a defined backlog, functions, and events. The Product Owner role was performed by a manager from the healthcare department, and development occurred in two-week sprints, including other SCRUM events such as planning, review, and retrospective meetings. These cycles allowed for continuous refinement of the architecture, incorporating technical and clinical feedback from the implementation environments, which directly influenced architectural decisions, rather than the evaluation of functional performance.

The architectural assessments were conducted in real-world operational environments, including state health secretariats, state regulatory centers, and referral hospital units in the states of Rio Grande do Norte, Espírito Santo, and Mato Grosso, as previously described. The evaluation process occurred continuously over six months to one year, depending on the state, involving face-to-face meetings, semi-structured interviews, and technical workshops, with the main objective of evaluating architectural adequacy, feasibility, and alignment with regulatory flows.

This characteristic of constant feedback is central to applied research of a constructive nature (design science research), in which the proposed artifact, which in this case is the technological architecture, is developed, evaluated, and adjusted in real-world operational contexts. Each evaluation cycle resulted in specific architectural refinements, such as the definition of interoperability mechanisms, traceability of regulatory flows, and integration between state systems, aiming to overcome the limitations identified in the previously used platform (SISREG), including the execution of usability tests for validation.

Therefore, the methodological process of this research was not limited to the construction of an information system to be implemented in different states of Brazil, but to the definition and validation of a reference architectural model capable of supporting interoperable, scalable, and transparent health regulation solutions, applicable to different contexts of the Unified Health System (SUS). The validation of the architecture was based on acceptance by the stakeholders involved, adherence to national health regulation guidelines, and operational feasibility observed in real-world implementation contexts, positioning the implemented system as an instance of the architecture, and not as the main object of evaluation, and demonstrating significant improvements compared to the previously adopted solution.

### 3.2 Survey and definition of architectural requirements

The survey of architectural requirements that guided the design of the healthcare regulation architecture was conducted collaboratively with managers and technical teams from the different state departments in the municipalities where it was implemented. This survey constituted a structured methodological stage aimed at informing architectural decision-making, and was carried out across different phases of implementation in a complementary manner, i.e., the nuances and difficulties identified in a given state were analyzed and adapted to inform architectural refinements applicable to other contexts.

In addition, this stage involved analyzing regulatory workflows, state and ministerial ordinances in force, clinical protocols, and interviews with professionals from regulation centers and health units. The main objective was to identify operational needs and technological gaps that compromised the efficiency and transparency of the regulatory process within the SUS, systematically translating these findings into architectural requirements—both functional and non-functional—that guided the design of the reference architecture.

The functional requirements arose from the need to improve integration between information systems, the traceability of requests, and the transparency of assistance queues. These requirements were interpreted at the architectural level, defining the need for interoperability with national and state platforms such as the National Registry of Health Establishments (CNES), the Procedure Table Management System (SIGTAP), the National Health Data Network (RNDS), and e-SUS, in order to ensure the exchange of structured data through standardized and secure interfaces. It should be noted that CNES maintains records of healthcare establishments, including their name, identification, location, number of employees, and specialties served. RNDS is the Ministry of Health platform responsible for interoperating and communicating health data from platforms used in Brazil. Finally, e-SUS is the system used to manage and classify primary care information, as well as to monitor patient health. In addition, architectural mechanisms were defined to enable centralized management of requests for beds, consultations, and specialized procedures, ensuring detailed records of each stage and a complete history of the actions performed.

Another essential requirement concerned real-time monitoring of bed occupancy and regulatory queues, allowing managers to identify bottlenecks, redistribute resources, and anticipate situations of healthcare overload. Finally, architectural provisions were established for the public availability of transparency dashboards, with anonymized data on waiting times and bed occupancy, as well as differentiated access control by user profile, including health managers, health professionals, and representatives of oversight bodies such as the Public Prosecutor’s Office and Courts of Auditors.

In addition to these aspects, non-functional requirements were defined for the robustness, interoperability, and security of the digital ecosystem. These requirements informed key architectural principles, including compatibility between different platforms and levels of care through the adoption of open interoperability standards such as HL7 FHIR, OAuth2, and OpenID Connect. Scalability and modularity were identified as fundamental principles to enable the incremental evolution of the architecture and its progressive adoption across heterogeneous regional contexts. The need for high availability and fault tolerance was also highlighted, ensuring service continuity even in situations of network overload or occasional infrastructure failures.

Compliance with the General Data Protection Law (Law No. 13,709/2018—LGPD) and with the information security guidelines of the Ministry of Health was defined as a mandatory requirement, directly influencing architectural decisions related to data governance, access control, and auditability, reinforcing the ethical and legal commitment to the protection of personal data.

Finally, the architecture should ensure full auditability of operations, recording detailed logs of all actions and promoting the traceability of regulatory decisions, in addition to offering high maintainability and reusability of its modules, as architectural qualities rather than system-specific features, in order to facilitate updates and upgrades without compromising the functioning of the ecosystem as a whole.

The consolidation of these requirements represented a fundamental milestone for the design of subsequent architectural decisions, guiding the choice of a layered architectural style and the adoption of interoperability standards. Thus, the proposed architecture is not restricted to the technical dimension of an information system, but constitutes a reference model for digital health governance, structured to respond to the demands of the SUS for integration, transparency, and regulatory efficiency.

### 3.3 Conceptual design and architectural style adopted

The technological architecture developed to support healthcare regulation was structured according to a distributed layered architectural model, defined as a reference architecture, composed of a modular backend layer and a decoupled frontend layer. This architectural choice was driven by the need to support interoperability, scalability, and governance across heterogeneous regulatory environments, as identified during the requirements survey within the SUS regulatory process. The backend layer was instantiated using the Django framework, while the frontend layer was designed to remain technologically independent, reinforcing architectural decoupling.

In addition to these two main layers, the architecture incorporates a set of auxiliary and specialized service layers, introduced to enhance operational efficiency without increasing coupling within the architectural core. Technologies such as MinIO and Celery were adopted to support external object storage and asynchronous processing flows, respectively. From an architectural perspective, these services were positioned as supporting components, enabling secure document management and background processing while preserving the stability, consistency, and governance of the core architecture.

The conceptual design of the architecture is grounded in established software engineering principles, including the application of the Adapter, Facade, and Template Method design patterns, as well as SOLID principles [21]. These patterns were deliberately selected to operationalize key architectural qualities, such as maintainability, extensibility, and interoperability, rather than to optimize isolated software components.

The Template Method pattern was applied to the architectural solution responsible for extracting and managing data from the SIGTAP table. At the architectural level, this pattern supports standardization and reuse in data integration workflows, defining a common abstraction through an abstract base class with nine methods responsible for extracting information from 16 SIGTAP tables. These tables are implemented through inheritance, requiring only one or two specific methods in each concrete class, thereby reducing architectural complexity, minimizing duplication, and facilitating long-term maintenance.

The Adapter pattern was employed in the integration with the Healthcare Regulation Information Registry (RIRA), a tool proposed by the SUS IT Department (DENASUS) to expand interoperability with the National Health Data Network (RNDS). According to its technical documentation, RIRA exposes RESTful web services based on the FHIR R4 standard [22, 23]. Within the proposed architecture, the Adapter pattern abstracts the complexity of this integration, allowing client components to interact with the service through a simplified interface and reducing the number of required parameters from 71 to 12. This decision illustrates how the architecture addresses interoperability challenges while preserving usability and consistency across integrations.This decision illustrates how the architecture addresses interoperability challenges while preserving usability and consistency across integrations.

The Facade pattern was applied to the integration of CADSUS (National Registry of Users of the Unified Health System) and RNDS to obtain updated individual health information. From an architectural standpoint, the Facade encapsulates the heterogeneity and complexity of external national systems, providing a unified access interface aligned with defined regulatory business rules. This approach reinforces architectural transparency and supports the governance requirements identified during the methodological stages of the research.

### 3.4 Development technologies

In order to meet the growing need for healthcare services, the architecture was designed to be used via the web, as a deployment strategy aligned with the architectural requirement of broad accessibility across heterogeneous healthcare contexts. Furthermore, in accordance with architectural principles described by Sommerville [24], the proposed reference architecture is structured into a visualization layer, a business logic layer, and a persistence and integration layer, with the Facade pattern supporting external system integrations.

For the visualization layer (frontend), Next.js was adopted as an instantiation technology of the architectural visualization layer. One of the main reasons for this choice was its scalability, as it combines server-side rendering with optimized content delivery, allowing applications to grow efficiently and adapt to increasing numbers of users and data processing demands. From an architectural standpoint, this choice supports reliability and responsiveness in public health platforms subject to access peaks, while remaining consistent with the decoupling principle defined in the reference architecture.

For the business logic and persistence layer (backend), the architectural components were instantiated using Django (with Django Ninja for APIs), combined with Celery for asynchronous processing, Redis as broker and cache, PostgreSQL as the database management system, and Daphne for WebSocket-based real-time communication. These technologies were selected to operationalize architectural qualities previously defined, rather than to prescribe a fixed technological stack.

Django was selected as a high-level Python-based framework due to its maturity, security mechanisms, and support for rapid development through a “batteries-included” approach. Within the context of the proposed architecture, Django supports the modularization of business rules and regulatory workflows, while Django Ninja facilitates the exposure of RESTful and OpenAPI-compliant interfaces, enabling standardized communication and automatic data validation.

For asynchronous activities and background tasks, such as notification dispatch and queue processing, Celery was adopted as a distributed task queue. Redis was used both as Celery’s message broker and as a caching mechanism, contributing to architectural requirements related to performance, responsiveness, and fault tolerance, particularly in scenarios with high read volumes and concurrent access.

PostgreSQL was chosen as the database management system due to its adherence to ACID properties, robustness in transactional processing, and advanced features such as support for JSON data types and geospatial queries. These characteristics align with the architectural need for consistency, auditability, and flexible data modeling in regulatory environments.

The requirement for real-time interaction between clients and servers, especially in monitoring and regulation workflows, motivated the use of Daphne as an ASGI server enabling WebSocket communication. At the architectural level, this decision supports low-latency data propagation for queue monitoring and operational dashboards, while communication between frontend and backend is conducted via REST APIs and WebSockets, with authentication based on JWT tokens. This combination reflects architectural trade-offs between simplicity, interoperability, and security, with opaque token-based authentication adopted to reduce exposure of sensitive data and ensure compliance with the General Data Protection Law (LGPD), enabling centralized session control and revocation.

### 3.5 Interoperability with healthcare systems

The regulatory architecture was designed to comprehensively address different modalities of access regulation in healthcare, with interoperability defined as a central architectural property rather than a system-level feature. The proposed architecture emphasizes scalability, maintainability, and adaptability, enabling continuous evolution and agile responses to changes in regulatory, organizational, and operational requirements within the public health system.

The architecture was organized into logically independent but interoperable architectural modules, which operate in an integrated manner to support a continuous flow of information across regulatory processes. These modules include hospital bed regulation, appointment scheduling, specialized procedure management, clinical treatment monitoring, and vascular care. From an architectural perspective, these modules represent separable regulatory domains, enabling independent evolution while preserving consistency and integration across the ecosystem. In addition, the architecture establishes standardized integration mechanisms with multiple databases and external systems maintained by the Ministry of Health—such as the National Register of Health Establishments (CNES), the Management System for the Table of Procedures, Medicines, and OPM (SIGTAP), and e-SUS—as well as with the National Health Data Network (RNDS), the Federal Council of Medicine (CFM), and the Brazilian Institute of Geography and Statistics (IBGE), ensuring interoperability across institutional and governmental boundaries.

Interoperability between architectural modules is a key element of the proposed model, enabling both internal communication among regulatory components and external communication with heterogeneous health information systems. This architectural approach facilitates standardized data exchange across different entities and levels of care, providing an integrated and auditable view of patient flows and available resources. By supporting interoperability at the architectural level, the proposed model addresses structural limitations of existing monolithic solutions and contributes to more efficient allocation and governance of healthcare resources within the SUS.

## 4 Results

As presented in the previous sections, the regulatory architecture was implemented in three different Brazilian states, each with unique characteristics in its access regulation processes. In this section, the results are presented from an architectural perspective, focusing on how the proposed reference architecture was instantiated and validated in real-world contexts, rather than on the evaluation of clinical or operational outcomes. Despite contextual differences, the implementations shared common architectural objectives, including support for health access management, information transparency, interoperability with the main systems of the Ministry of Health, and controlled access by regulatory and oversight institutions.

Furthermore, the results reflect the architectural properties achieved through the proposed model, such as integrity, interoperability, and support for equitable regulatory workflows across heterogeneous settings. These results are evidenced by the materialization of architectural decisions that enable the management of beds, exams, regulatory queues, vascular care lines, and dialysis treatments within a unified and auditable digital ecosystem. The proposed architecture also demonstrated the capacity to accommodate future extensions, reinforcing its role as a scalable and adaptable reference model for healthcare regulation.

### 4.1 The regulatory architecture

Within the context of software engineering, architecture defines the components of a system, their interactions, and their relationships with the external environment [25]. In this study, the architectural perspective is adopted as the primary analytical lens to present the results, as it enables a systematic understanding of structural decisions, responsibility allocation among components, and interaction patterns that support integrity, interoperability, and governance within the healthcare regulation domain.

There are different approaches for representing software architectures, such as UML diagrams and the 4+1 model. However, to ensure clarity, traceability, and consistency in the presentation of the architectural results, this study adopts the C4 model proposed by Brown [26]. The C4 model was selected as a methodological instrument to explicitly represent the proposed reference architecture at multiple abstraction levels, encompassing context, containers, and components.

The first level of representation corresponds to the context layer, which provides a high-level view of the regulatory ecosystem and its interactions. At this level, the result highlights how the proposed architecture positions itself within the broader health information ecosystem, identifying key user profiles—Health Manager, Health Professional, and Patient—and their interaction with the regulatory interface responsible for request submission and regulatory management.

In addition, essential external systems are represented, such as CAD-SUS, SIGTAP, RIRA/RNDS, the Federal Council of Medicine (CFM), IBGE, CNES, Gov.br, MinIO, and Sabiá. From an architectural standpoint, these systems are treated as external dependencies integrated through standardized interfaces, illustrating the interoperability mechanisms defined in the architectural design rather than specific implementation details.

**Figure 1:**
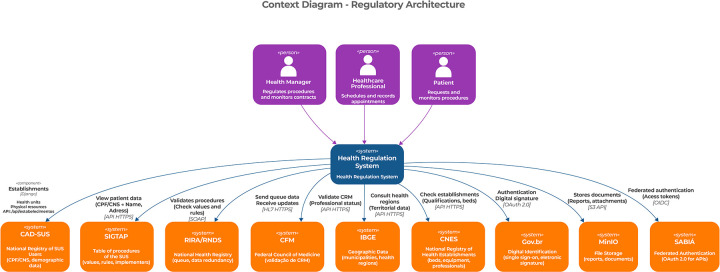
Presentation of the architecture context layer.

The relationships between the regulatory interface, users, and external systems are described through connections that indicate information flows and communication standards, including RESTful APIs over HTTPS, SOAP protocols, HL7 FHIR for clinical data exchange, OAuth 2.0 and OpenID Connect (OIDC) for authentication, and S3-compatible interfaces for object storage. These relationships constitute architectural evidence of how interoperability and secure integration were operationalized. Figure 1 presents the context layer of the proposed regulation architecture.

The second level of the C4 model corresponds to the container layer, which decomposes the architecture into major execution units and illustrates how responsibilities are distributed across them. This level represents an architectural result by demonstrating how the proposed architecture supports scalability, separation of concerns, and controlled interaction between layers. In this view, actors interact with the “Web Application” container, which represents the visualization layer of the architecture and is instantiated using Next.js technologies to support responsive and scalable user interfaces.

This container connects to the “Web Server” container, represented by Nginx, which performs load balancing, SSL termination, and request routing. From an architectural perspective, this component acts as an intermediary that enforces communication policies between frontend and backend layers. Nginx forwards requests to the application containers responsible for synchronous and asynchronous processing, including the “ASGI App Server” and the “WSGI App Server for WebSocket,” which together support RESTful services and real-time communication.

**Figure 2:**
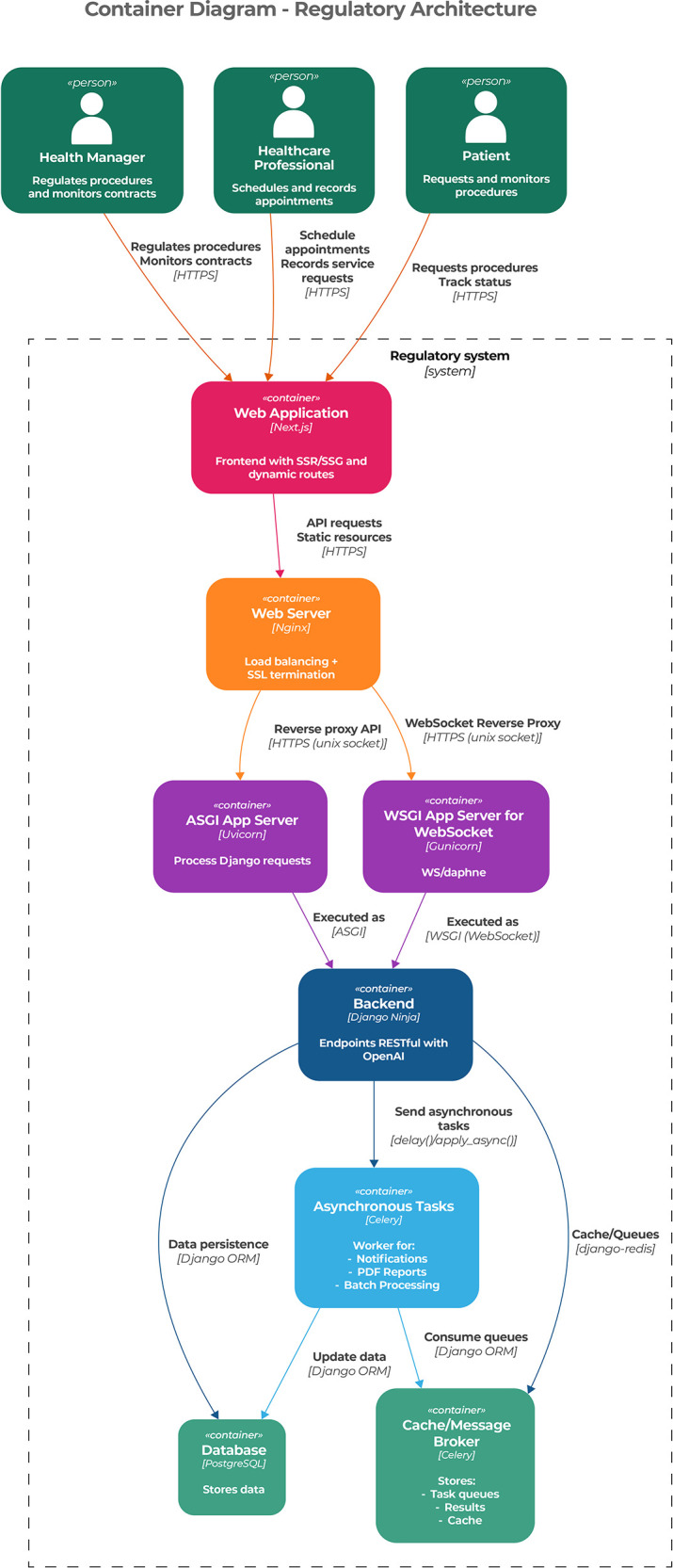
Presentation of the container layer of the architecture.

The core backend logic is represented by the “Backend” container, which exposes RESTful APIs documented through OpenAPI and interacts with auxiliary containers for asynchronous tasks, persistence, and caching. These containers illustrate how the architecture separates core regulatory logic from supporting services, such as background task execution, data persistence, and message brokering, thereby reinforcing modularity and maintainability. Figure 2 presents the container layer of the proposed architecture.

The third level of the C4 model corresponds to the component layer, which details the internal structure of the backend container. At this level, the results demonstrate how regulatory responsibilities are decomposed into coherent architectural components, including Basic Records, Establishments, Financial Management, Regulation, Authentication, People Management, Requests, Scheduling, Notifications, Procedures, and Facilities.

**Figure 3:**
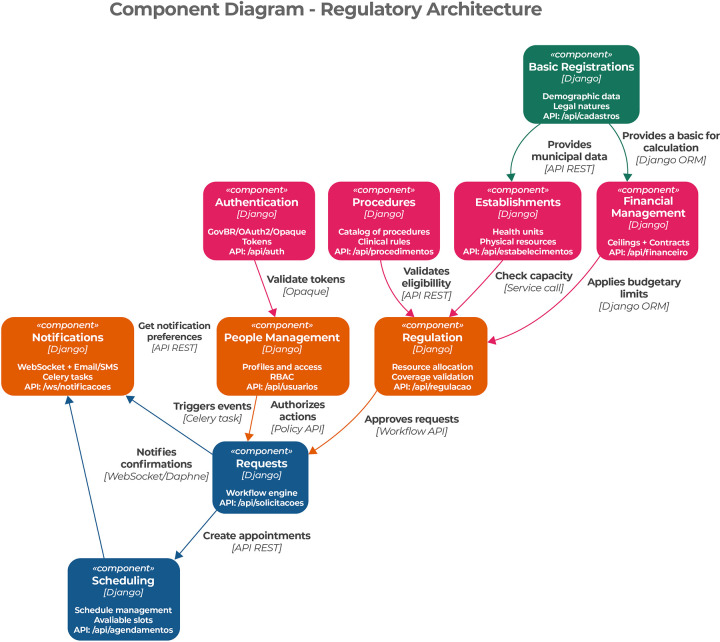
Presentation of the architecture component layer.

The interactions among these components illustrate how the architecture enforces regulatory workflows, access control, and validation rules in an integrated manner. This component-level view provides architectural evidence of how traceability, governance, and interoperability requirements are addressed, as regulatory decisions are supported by coordinated interactions among clinical, administrative, and infrastructural components. Figure 3 presents the component layer of the proposed architecture.

### 4.2 Transparency in bed management

**Figure 4:**
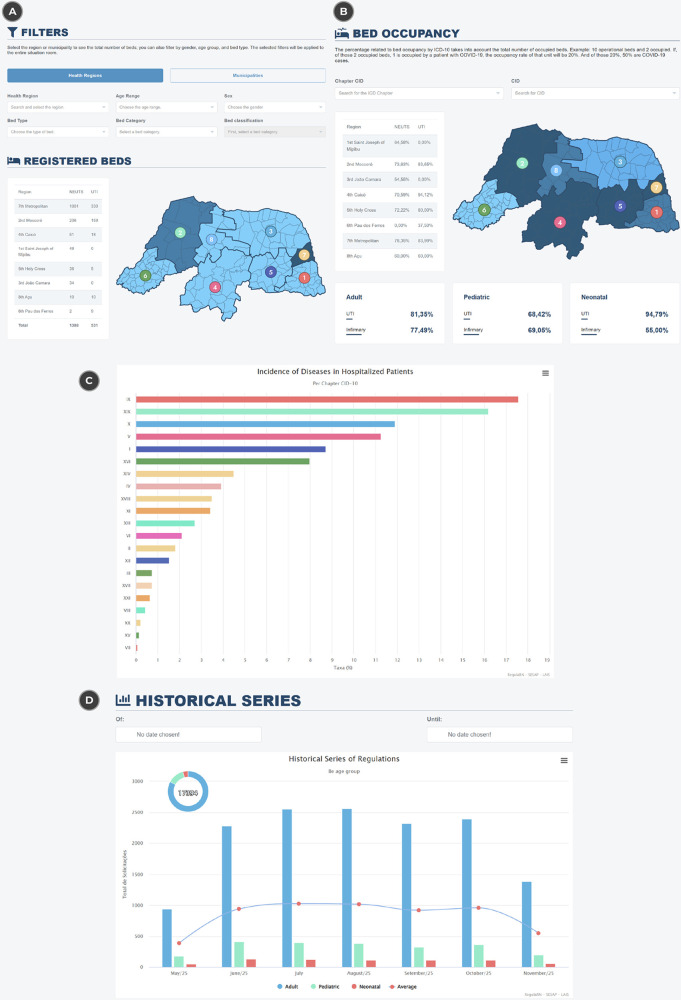
Presentation of the information panel to represent bed regulation data. (**A**) The number of requests by region, (**B**) occupancy rates by type (adult, pediatric and neonatal), (**C**) the incidence of diseases in hospitals and (**D**) a historical series of requests divided by groups. Reproduced with permission from “Situation Room Beds” by the Laboratory of Technological Innovation in Health and the State Secretariat of Public Health of Rio Grande do Norte.

One of the notable results of the proposed architecture is the architectural support for transparency in the management of waiting lists, which constitutes a fundamental requirement for equitable and accountable regulation of health resources. At the architectural level, this transparency is achieved by enabling the structured visualization and aggregation of regulatory data, including the number of ICU beds and wards registered by health region or municipality, age group, gender, bed type, category, and classification. These representations demonstrate the architecture’s ability to consolidate heterogeneous data sources into a unified and auditable view, as illustrated in Figure 4A.

In addition to the registration of available beds, the architecture enables the real-time calculation and visualization of bed occupancy rates, which constitutes an important architectural capability for regulatory monitoring. This capability supports situational awareness regarding demand and capacity, allowing regulators to observe occupancy levels by bed type (ICU or ward) and subclassifications such as adult, pediatric, or neonatal, as shown in Figure 4B.

From an architectural perspective, the availability of occupancy indicators illustrates how the proposed model supports informed regulatory oversight, without prescribing specific decision-making strategies. These indicators exemplify the type of regulatory information that can be derived from the architectural design, enabling managers to observe patterns such as sustained high occupancy or underutilization of resources.

The distribution of the most prevalent diseases is also presented as an architectural outcome related to data integration and traceability. By enabling the aggregation and visualization of clinical and regulatory data, the architecture supports the identification of disease incidence patterns among hospitalized patients, as illustrated in Figure 4C. This functionality demonstrates the capacity of the architecture to integrate clinical and regulatory dimensions, rather than constituting an evaluation of epidemiological outcomes.

Another relevant architectural result is the support for temporal analysis of hospitalization requests, enabled through the visualization of historical series categorized by bed type. This capability illustrates how the architecture accommodates longitudinal regulatory data, allowing users to explore trends and seasonal variations across different time periods, as shown in Figure 4D.

It should be noted that the presented visualizations exemplify the types of indicators enabled by the proposed architecture, reflecting regulatory information commonly required by healthcare units and public health institutions for monitoring and oversight. Due to the modular and flexible nature of the architecture, additional representations and indicators can be incorporated, adapting the model to the specific needs of different regions. Furthermore, all data used to generate the presented panels were anonymized in compliance with the Brazilian General Data Protection Law, and the architecture supports secure data access and controlled data export, reinforcing its alignment with legal and ethical requirements.

### 4.3 Transparency in outpatient care management

In the context of outpatient care management, the proposed regulatory architecture supports transparency by enabling structured visualization and filtering of regulatory data related to outpatient procedures and specialties. At the architectural level, this transparency is achieved through the integration and aggregation of data that can be filtered by procedure or specialty type, subgroup, procedure or specialty name, sedation requirement, care segment, health region, requesting municipality, and time period.

**Figure 5:**
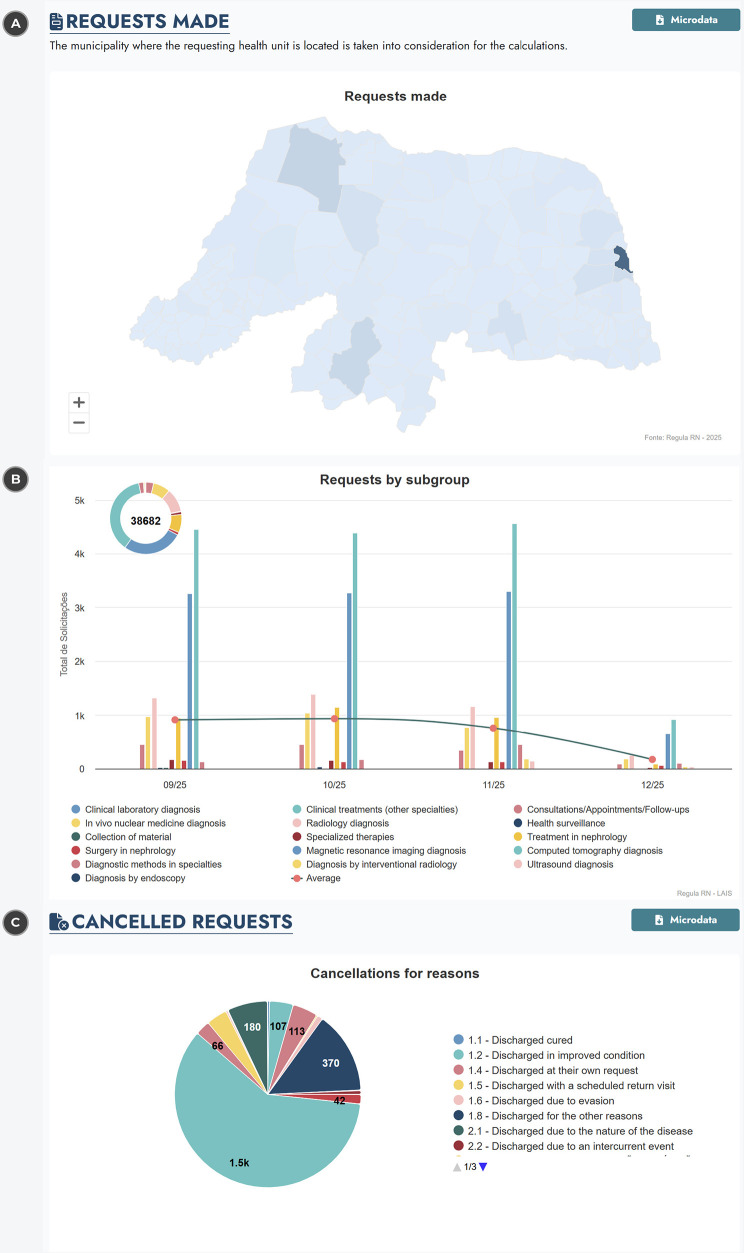
Presentation of the information panel to represent the data on outpatient patient regulations. (**A**) The number of requests by municipality, (**B**) the number of requests by care groups and (**C**) the requests canceled by reason. Reproduced with permission “Situation Room Regula RN” by the Laboratory of Technological Innovation in Health and the State Secretariat of Public Health of Rio Grande do Norte.

These filtering capabilities demonstrate the architecture’s ability to organize outpatient demand information in a granular and auditable manner, allowing users to explore how requests are distributed across territories and specialties. Figure 5A illustrates the visualization of municipalities responsible for requesting a selected procedure or specialty, while Figure 5B exemplifies the grouping of requests by subgroup, highlighting the architecture’s support for demand categorization.

Another relevant architectural result is the support for monitoring and analyzing canceled outpatient requests, which reflects the system’s capacity for traceability and accountability throughout the regulatory workflow. The architecture enables the classification and visualization of cancellations by reason and by requester, as shown in Figure 5C, providing a transparent record of regulatory events rather than an interpretation of their causes.

From an architectural standpoint, the availability of cancellation-related indicators demonstrates the model’s ability to preserve historical regulatory data and support auditing processes, enabling health managers and oversight institutions to examine outpatient regulation flows. Due to the modular nature of the architecture, additional outpatient indicators and analytical perspectives can be incorporated, allowing adaptation to the specific informational needs of different regulatory contexts.

### 4.4 Transparency in vascular care management

The proposed regulatory architecture also supports transparency in the management of the vascular care pathway, enabling the structured organization and visualization of information related to patients with vascular diseases. From an architectural perspective, this functionality is achieved through the consolidation of procedural and regulatory data associated with the vascular care line, allowing different analytical views to be generated from the same data source.

**Figure 6:**
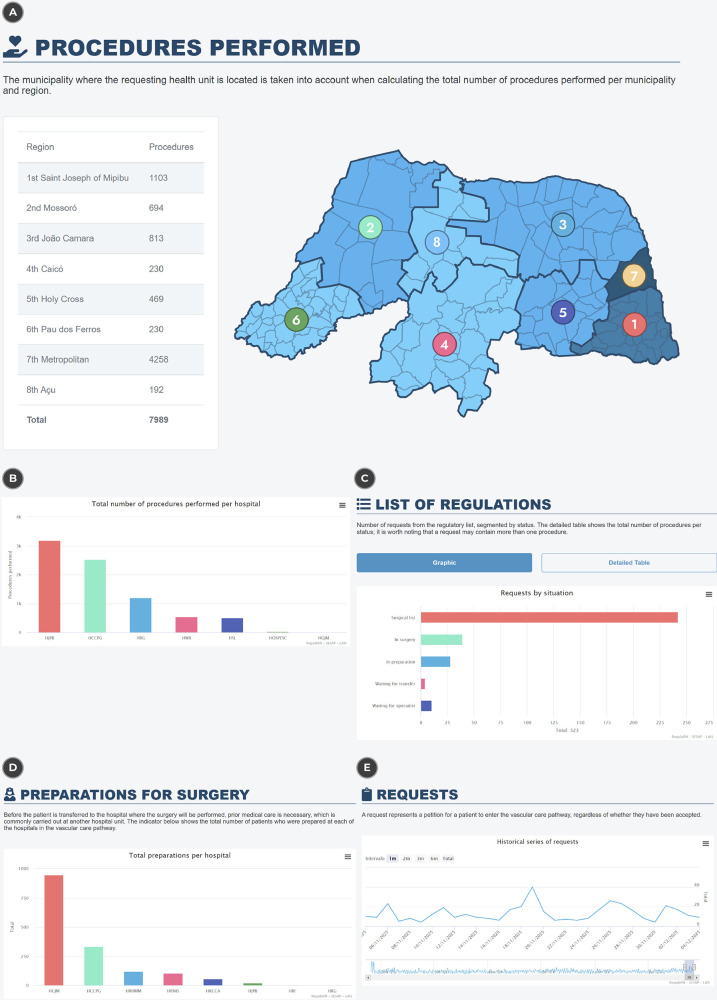
Presentation of the information panel to represent vascular regulation data. (**A**) The number of procedures per region, (**B**) the number of procedures per hospital unit, (**C**) the list of requests in the regulation queues, (**D**) the number of requests in preparation for surgery and (**E**) the historical series of requests. Reproduced with permission from “Vascular Care Pathway Situation Room” by the Laboratory of Technological Innovation in Health and the State Secretariat of Public Health of Rio Grande do Norte.

Within the transparency reports, the architecture enables analysis of the total number of vascular procedures performed, with filters by health region, municipality, and performing hospital. These visualizations demonstrate the system’s capability to represent the spatial and institutional distribution of vascular procedures, supporting comparative analyses across regions and healthcare facilities. Figure 6A,B present, respectively, the distribution of procedures by region and the total number of procedures performed per hospital.

Another relevant result of the architecture is the ability to monitor the regulatory status of vascular care requests throughout the care pathway. The system records and displays the number of requests according to predefined regulatory situations, such as surgical list, in surgery, preparation, awaiting transfer, and awaiting specialist. This classification reflects the architecture’s support for workflow tracking and status traceability, which can be presented in graphical or tabular formats, as illustrated in Figure 6C.

The architecture also records and makes visible the preparatory phase preceding surgical interventions, in which patients receive prior medical care at designated hospitals before transfer for surgery. This functionality highlights the system’s capacity to document intermediate stages of the care pathway, as shown in Figure 6D, which presents the number of patients prepared in each hospital within the vascular care line.

Finally, the architecture provides access to historical series of vascular care requests, distinguishing between accepted and rejected cases over time. This temporal visualization demonstrates the system’s ability to preserve historical regulatory data and support longitudinal analyses of request volumes, as illustrated in Figure 6E.

### 4.5 Queue management

One of the main results of the proposed architecture is the formalization and structuring of queue management mechanisms for hospital beds, outpatient care, and vascular care. From an architectural standpoint, queue management is implemented through explicit prioritization rules, standardized workflows, and persistent state control, allowing regulatory queues to be managed according to predefined and auditable criteria. These mechanisms ensure that the ordering of requests follows transparent and traceable rules, preventing unauthorized changes to priority levels or request positions.

All queue-related operations are fully logged by the system, including changes in priority, status updates, and decision justifications. This auditability allows regulatory actions to be reconstructed and inspected, strengthening institutional oversight and enabling verification by management bodies and external control institutions. In this sense, queue management emerges not as an operational outcome, but as a structural property of the architecture, aligned with the requirements of transparency and accountability in the SUS.

Furthermore, the architecture supports the organization of care flows by integrating real-time operational data with formal regulatory protocols. The system allows the configuration and application of prioritization models, such as the Unified Prioritization Score and the SOFA (Sequential Organ Failure Assessment), which are used as rule-based inputs within the regulatory workflow, ensuring that clinical severity criteria are consistently applied during queue ordering.

Rather than enforcing policy decisions, the architecture provides the technical means to operationalize existing regulatory protocols, enabling consistent prioritization across different care modalities. As a result, queue management is supported by a combination of traceable decision rules, interoperable clinical data, and auditable workflows, reinforcing the architecture’s role as a reference model for transparent and accountable regulation of access to health services.

### 4.6 Access by public control agencies

It is worth highlighting the architectural provision of differentiated access mechanisms for public oversight institutions, such as the Public Prosecutor’s Office (MP), the Office of the Comptroller General (CGU), the Federal Court of Accounts (TCU), and State and Municipal Courts of Accounts (TCEs and TCMs). The proposed architecture explicitly incorporates role-based access control (RBAC) profiles that allow these institutions to consult regulatory data related to waiting lists and regulatory workflows, including request status, prioritization criteria, and historical decision records.

Through this dedicated access, oversight institutions are able to retrieve structured and up-to-date information directly from the regulatory system, without interference in operational workflows. This architectural feature enables external inspection of regulatory processes, supporting the monitoring of compliance with predefined rules, prioritization protocols, and governance guidelines established within the SUS.

This differentiated access model represents an architectural advance in terms of digital governance, as it embeds transparency and auditability as first-class design requirements rather than post hoc reporting mechanisms. By exposing regulatory data through controlled and read-only access profiles, the architecture supports inspection, supervision, and accountability processes while preserving system integrity and operational security.

Thus, the availability of structured regulatory data to public control agencies is not presented as an outcome of system use, but as a design property of the architecture itself, reinforcing its suitability as a reference model for transparent, auditable, and institutionally accountable healthcare regulation within the SUS.

## 5 Discussions

A regulatory architecture represents an important advance from a design, governance, and organizational perspective in the management of the Brazilian public health system. Rather than focusing on the measurement of clinical or operational outcomes, the contribution of this study lies in the definition of a reference architectural model capable of structurally supporting health regulation processes, including real-time data availability, interoperability with municipal, state, and federal information systems, and transparency and auditability requirements. The proposed architecture establishes technical and organizational mechanisms intended to support equitable, rule-based allocation of healthcare resources, reducing discretionary practices and increasing institutional accountability.

The analysis of the implementation contexts indicates that an interoperable architectural model, anchored in open standards such as HL7 FHIR (R4) and the National Health Data Network (RNDS), and supported by established software engineering practices (including the C4 architectural model and design patterns), contributes to reducing informational fragmentation and coordination barriers in the regulatory cycle of request submission, prioritization, and resource allocation. These architectural properties facilitate structured data exchange, traceability of regulatory decisions, and integration across levels of care, aligning the proposed model with the requirements of public administration and digital health governance.

When compared to the regulatory system currently adopted at the national level, SISREG, the proposed architecture represents a substantive conceptual and technical shift. SISREG was designed as a centralized and highly coupled monolithic system, whose architectural conception reflects the technological constraints of the mid-2000s. Furthermore, DATASUS does not provide any document that explicitly and transparently details its architecture, nor does it indicate that SISREG follows any international communication standard for health data. As a result, it offers limited native support for modular evolution, interoperability with heterogeneous state-level platforms, real-time monitoring, and comprehensive auditability. In contrast, the architecture proposed in this study was conceived as a distributed, layered, and modular reference model, explicitly designed to address these structural limitations. By incorporating an explicit interoperability layer, adopting open standards, and embedding traceability, auditability, and role-based access control as first-class architectural requirements, the proposed model enables integration across decentralized healthcare systems while preserving local autonomy and regulatory diversity.

Regarding data consolidation and availability, the proposed architecture enables the integration of heterogeneous regulatory domains—including bed management, outpatient care, examinations, and vascular procedures—into a unified digital ecosystem. This integration supports managerial analysis and planning by allowing stakeholders to visualize demand, capacity, and regulatory flows in a structured and consistent manner. These capabilities should be understood as architectural affordances rather than measured performance gains, as they establish the technical conditions required for informed decision-making, adaptive planning, and coordinated action within complex healthcare networks such as the SUS.

In terms of public transparency, the architecture explicitly embeds transparency and auditability as core design principles rather than secondary reporting features. By providing controlled, read-only access profiles for citizens and public oversight institutions, such as the Public Prosecutor’s Office, the Office of the Comptroller General, and Courts of Accounts, the model supports external inspection and accountability while preserving operational security and data integrity. This architectural choice aligns with contemporary governance frameworks that emphasize traceability, institutional trust, and social control in public administration [27–29].

With respect to queue management, the proposed architecture was designed to accommodate multiple prioritization and regulation models, allowing the incorporation of existing local criteria, ordinal priority scales, and formal clinical scores such as the Sequential Organ Failure Assessment (SOFA) and the Unified Prioritization Score (EUP). The architecture itself does not define or validate the clinical effectiveness of these models; instead, it provides a flexible and extensible technical structure capable of operationalizing diverse regulatory policies as defined by health authorities. Previous studies reporting the effectiveness of such prioritization strategies [4, 30] are referenced to demonstrate conceptual alignment with established regulatory practices, rather than as a direct evaluation of the proposed architectural model. It is noteworthy that the proposed architecture does not rely on opaque or fully automated decision-making mechanisms. Prioritization rules and clinical scores are defined by public health authorities and incorporated as explicit, transparent, and auditable protocols, while final regulatory decisions remain under human oversight and institutional accountability.

It is also important to highlight that the architecture was instantiated in high-pressure operational contexts, including its use as part of a broader digital ecosystem during the COVID-19 pandemic in the state of Rio Grande do Norte [29]. This context exposed the architecture to significant scalability, interoperability, and real-time data demands, reinforcing its suitability as a reference model for dynamic and crisis-sensitive regulatory environments [18, 31, 32]. However, no formal impact assessments, usability studies, or controlled experiments were conducted. Therefore, the observations reported in this study are limited to architectural feasibility, operational viability, and stakeholder acceptance, rather than demonstrable improvements in efficiency, waiting times, health outcomes or user evaluation. This limitation could be addressed in future work using TAM, TOE, or UTAUT-based evaluation techniques.

Despite the architectural contributions presented, this study is subject to important limitations inherent to the development and validation of complex sociotechnical systems in public healthcare environments. One of the main challenges encountered was the elicitation and consolidation of architectural requirements in contexts characterized by heterogeneous stakeholders. Divergent priorities, institutional interests, and operational practices among health managers, regulatory teams, clinicians, and technical staff required iterative negotiation and compromise, influencing both the pace and scope of architectural decisions.

Another relevant limitation concerns user acceptance, particularly among some physicians, nurses, and technical staff. Resistance to the adoption of the proposed platform was observed in certain contexts, especially when the system introduced changes to established workflows, increased traceability of regulatory decisions, or required stricter adherence to formal protocols. These challenges are consistent with findings in the literature on health information systems and reflect organizational, cultural, and human factors rather than technical shortcomings of the architecture itself.

Finally, the main contribution of this work lies in the definition and validation of a flexible, layered reference architecture for healthcare regulation, designed to be articulated with contemporary software technologies and national interoperability platforms. By explicitly incorporating interoperability mechanisms, governance structures, and auditability requirements, the proposed model addresses long-standing challenges related to information fragmentation, limited transparency, and coordination in decentralized healthcare systems such as the SUS. As a reference architecture, its purpose is to inform, guide, and support future implementations and adaptations, rather than to report the outcomes of a single system deployment or to claim direct causal impacts on healthcare delivery.

## 6 Conclusions

The technological regulatory architecture presented in this study demonstrates that robust, interoperable, and auditable digital architectures are essential to structurally support health governance within the Unified Health System (SUS). Rather than claiming measured operational or clinical impacts, the proposed model establishes a reference architectural framework capable of integrating heterogeneous data sources, structuring transparent regulatory workflows, and enabling traceability and real-time inspection of regulatory decisions. These characteristics position the architecture as a technical foundation aligned with the governance, accountability, and interoperability requirements of contemporary public healthcare systems.

The instantiation of the architecture in three Brazilian states, Rio Grande do Norte, Espírito Santo, and Mato Grosso, illustrates its flexibility and adaptability across heterogeneous organizational and institutional contexts. This diversity of implementation environments reinforces the suitability of the proposed model as a reference architecture, capable of supporting decentralized regulatory arrangements while preserving local autonomy and alignment with national health policies. Importantly, these implementations serve as evidence of architectural feasibility and operational viability, rather than as formal evaluations of system performance or health outcomes.

The main contributions of this work reside in the definition of a layered, modular, and interoperable architectural model that embeds transparency, auditability, and governance mechanisms as first-class design principles. By supporting transparent queue management, integration across levels of care, and controlled access for public oversight institutions, the architecture provides a structured digital foundation for regulatory processes in the SUS. In this sense, the proposed model should be understood as a strategic architectural benchmark for digital health initiatives, intended to guide future system designs and adaptations rather than to replace existing regulatory policies or to claim direct causal effects on healthcare delivery.

Future research may build upon this reference architecture by conducting formal usability studies, quantitative performance evaluations, and longitudinal analyses of regulatory outcomes, thereby extending the architectural contribution presented here toward empirical assessment of its impacts in diverse healthcare settings.

## Data Availability

The raw data supporting the conclusions of this article will be made available by the authors, without undue reservation.
